# Qualitative assessment of attributes and ease of use of the ELLIPTA™ dry powder inhaler for delivery of maintenance therapy for asthma and COPD

**DOI:** 10.1186/1471-2466-13-72

**Published:** 2013-12-07

**Authors:** Henrik Svedsater, Peter Dale, Karl Garrill, Richard Walker, Mark W Woepse

**Affiliations:** 1 Global Health Outcomes, GlaxoSmithKline, Stockley Park, Uxbridge, UK; 2 HEOR Solutions, London, UK; 3 Medicine and Process Delivery, GlaxoSmithKline, Stockley Park, Uxbridge, UK; 4 Product Development, GlaxoSmithKline, Stockley Park, Uxbridge, UK; 5 Strategic Eye, Inc., 631 Thomas Jefferson Road, Wayne, PA, USA

**Keywords:** Ease of use, ELLIPTA inhaler, Inhaled therapy, Inhaler preference, Patient interviews

## Abstract

**Background:**

Medications for respiratory disorders including asthma and chronic obstructive pulmonary disease (COPD) are typically delivered to the lung by means of a handheld inhaler. Patient preference for and ability to use the inhaler may influence their adherence to maintenance therapy, and adherence may affect treatment outcomes. In this study, patient experience of using a dry powder inhaler (DPI), the ELLIPTA™ DPI, in clinical trials of a new maintenance therapy for asthma and COPD was investigated. The ELLIPTA DPI has been designed to contain two separate blister strips from which inhalation powder can be delivered, and to be simple to use with a large, easy-to-read dose counter.

**Methods:**

Semi-structured, in-depth, qualitative interviews were carried out 2–4 weeks after patients had completed one of six phase IIIa clinical trials using the ELLIPTA DPI. Interview participants were asked about their satisfaction with various attributes of the inhaler and their preference for the ELLIPTA DPI relative to currently-prescribed inhalers, and responses were explored using an inductive content analysis approach. Participants also rated the performance of the inhaler on several criteria, using a subjective 1–10 scale.

**Results:**

Participants with asthma (n = 33) and COPD (n = 42) reported high levels of satisfaction with the ELLIPTA DPI. It was frequently described as straightforward to operate and easy to use by interview participants. Ergonomic design, mouthpiece fit, and dose counter visibility and ease of interpretation emerged as frequently cited drivers of preference for the ELLIPTA DPI compared with their current prescribed inhaler. Of participants with asthma, 71% preferred the ELLIPTA DPI to DISKUS™ and 60% to metered dose inhalers. Of participants with COPD, 86% preferred the ELLIPTA DPI to DISKUS, 95% to HandiHaler™, and 85% to metered dose inhalers. Overall average performance scores were >9 (out of 10) in participants with asthma and COPD.

**Conclusion:**

The ELLIPTA DPI was associated with high patient satisfaction and was preferred to other inhalers by interview participants with asthma and COPD. The development of an inhaler that is regarded as easy and intuitive to use may have positive implications for adherence to therapy in asthma and COPD.

**Trial registration:**

Asthma: NCT01165138, NCT01431950. COPD: NCT01053988, NCT01054885, NCT01009463, NCT01017952.

## Background

Asthma and chronic obstructive pulmonary disease (COPD) are chronic respiratory disorders associated with significant morbidity and are commonly treated with inhaled therapies. Despite the availability of effective therapies, asthma remains uncontrolled in many patients [1]. COPD is a significant cause of both morbidity and mortality in later life, the burden of which is predicted to increase in aging populations [2]. Suboptimal adherence to maintenance therapies in asthma [3] and COPD [4] increases the burden of these diseases on patients and healthcare systems.

Maintenance therapy, consisting of an inhaled corticosteroid (ICS) alone or in combination with a long-acting β_2_ agonist (LABA) is recommended for patients whose asthma is uncontrolled on ICS alone [5]. ICS/LABA therapy is recommended for patients with moderate-to-severe COPD and/or history of COPD exacerbation [6]. The inhaler used to deliver maintenance therapies has a recognised effect on the effectiveness of therapy [7,8]. Patient satisfaction with the inhaler used to deliver their medication [9], and their competence in its use [10], have been identified as modifiable factors that may influence adherence to prescribed treatment. In an analysis of data from the 3-year TORCH study in COPD, a significant association was found between low (<80%) adherence, mortality, and COPD-related hospital admission [11]. In asthma, non-adherence to maintenance therapies is common and may be a factor contributing to poor asthma control [12].

The ICS fluticasone furoate (FF) has been developed as a monotherapy for asthma, and in combination with the LABA vilanterol (VI) as a once-daily inhaled maintenance therapy for asthma and COPD. Currently available ICS/LABA combination therapies require twice-daily dosing. By simplifying the dosing schedule, a shift from twice-daily to once-daily treatment may have a beneficial effect on treatment adherence [13,14]. However, a range of other factors are hypothesised to contribute to treatment adherence in asthma [15] and COPD [16]. FF/VI is delivered via a new dry powder inhaler (DPI), the ELLIPTA™ DPI. The inhaler is not yet in use in clinical practice and as such has no real-world test experience. In view of the potential effects of the perception of the delivery device on treatment adherence [8,17], we considered it to be clinically important to gain a better understanding of patients’ attitudes towards the ELLIPTA DPI.

To this end, an exploratory, qualitative study was performed in a subset of patients with asthma and COPD who participated in phase III trials of FF/VI or FF monotherapy and were subsequently interviewed about their experience of using the ELLIPTA device. Through conducting this study using an inductive methodology, we aimed to identify any hitherto unanticipated patient-perceived issues with the ELLIPTA DPI, and to gain an understanding of the circumstances under which those issues arose. We also sought information around patients’ thoughts on specific attributes of the inhaler and comparative preference relative to currently-prescribed alternative inhalers.

## Methods

### Study design

Patients who completed all study and follow-up visits in the following phase III trials of FF/VI or FF in COPD: HZC112206 [ClinicalTrials.gov: NCT01053988], HZC112207 [NCT01054885], HZC102871 [NCT01009463], HZC102970 [NCT01017952]; or asthma: HZA106827 [NCT01165138], FFA114496 [NCT01431950], were eligible to enter the qualitative study. Patients gave informed consent to participate in this qualitative study at the final or penultimate scheduled clinic visit. One-on-one in-depth interviews were conducted by telephone in order to gather information on participants’ perception of and satisfaction with the ELLIPTA DPI. To ensure that the patients would be able to accurately recall their experience with the ELLIPTA DPI, the interview was conducted within 4 weeks of the individual participant completing all trial procedures. Interview participants were paid a fee of US $100 to compensate them for the time and inconvenience associated with study participation.

### Participants

All interview participants were recruited from study sites in the USA (asthma: CA, FL, OR; COPD: AL, FL, IN, NC, OH, OR, SC, TX), were ≥18 years of age, had English as their primary language, could read English to an acceptable level, did not have any hearing impairment that could affect the interview, and had high-speed internet access or could receive express mail materials to aid discussion as necessary. Patients meeting these eligibility criteria and completing one of the clinical studies listed above within the recruitment time period were approached regarding participation in the interview-based study. The recruitment sample was thereby determined by the numbers of patients at eligible study sites who completed one of these clinical studies and consented to participate in interviews. Further details of the clinical studies from which participants were recruited, including the primary endpoints of the studies, are provided in Additional file 1: Table S1.

### Qualitative interviews

Qualitative interviews followed a semi-structured format based upon a discussion guide (Additional file 2) and focused only on the participant’s experience with the ELLIPTA DPI during the clinical study; participants were not asked about the study treatments. The discussion guide was developed to build on exploratory qualitative work with asthma and COPD patients and physicians, and was approved as part of the protocol. Interviews were approximately 45 min in duration and consisted of three parts:

1) Introduction: general questions about the participant’s asthma or COPD and their previous experience of inhaler use. Patients were also asked to describe their disease severity on a 10-point numerical rating scale [18] (1 = not at all severe, 10 = extremely severe).

2) Inhaler discussion: questions focusing on the participant’s perception of the ELLIPTA DPI, its ease of use (including their impressions of the instructions provided with the inhaler), storage, transportation, and the participant’s preference for the ELLIPTA DPI relative to their currently prescribed inhaler. During this part of the interview, participants were asked to evaluate the ELLIPTA DPI, on a 10-point scale (1 = worst, 10 = best), with respect to several key performance measures.

3) Follow-up questions/probes: exploring specific issues or queries that had previously been identified, or had arisen earlier in the interview.

Visual stimuli were used as prompts during the interviews (Additional file 3): specifically, images of the plain, unbranded ELLIPTA DPI used in the clinical trials, the patient instructions, and illustrations of ways in which users had been observed to open and grip the DPI. These images were posted on a secure website to which login details were provided to the participant in advance of the interview session, or express-mailed with instructions not to open sealed labelled materials until instructed by the interviewer.

The interview-based study was conducted by Strategic Eye, Inc. on behalf of GlaxoSmithKline. Interviews were conducted by two researchers employed by Strategic Eye. Strategic Eye worked directly with the clinical study sites to arrange interviews with patients who completed the clinical trial and provided informed consent to participate in interviews. All staff involved in the conduct of the study maintained confidentiality of all collected data, and any identifiable patient information was erased by Strategic Eye prior to providing interview reports to GlaxoSmithKline. The study protocol was reviewed and approved by Schulman Associates Institutional Review Board (Cincinnati, United States).

### Data analysis

Responses to open-ended questions were transcribed and an inductive content analysis approach [19] was applied to the transcripts to identify the concepts and ideas underlying the comparative preferences and patient perceptions of inhaler attributes. For each set of responses upon which the content analysis was conducted, an open coding process was used to determine relevant emerging themes and subsequently identify clusters within these themes. Additionally, a qualitative descriptive analysis technique [20] was used to analyze narrative descriptions of events, such as those provided by patients who described instances of incorrect use of the device. The use of these methods aided the researchers in understanding human elements of device use in a way that would not be identified via a pre-planned hypothesis testing approach.

Numerical data were generated from responses to closed questions regarding inhaler attributes and preference. No statistical inference was planned or applied to these data, which are presented in summary form for descriptive interpretation only within the context of a qualitative and exploratory study.

## Results

### Participant demographics

A summary of demographic data for participants with asthma and COPD is provided in Table 1. Participants with COPD were typically older than those with asthma and more likely to suffer from co-morbid conditions, such as arthritis, and to be using multiple medications each day. Participants with asthma reported longer duration of disease since diagnosis, and correspondingly longer and more varied history of inhaler use. Participant-reported severity of disease was on average moderate (5.0–5.6/10) for both asthma and COPD.

**Table 1:** Patient demographics

	**Asthma population**	**COPD population**
	**(n = 33)**	**(n = 42)**
Age, years	41	61
Duration of disease, years	22.1	7.1
Self-reported disease severity, 1–10 scale*	5.0	5.6
**Current inhaler**^**†**^		
DISKUS only, n	21	11
HandiHaler and DISKUS, n	N/A	10
HandiHaler only, n	N/A	10
MDI/HFA, n	10	11
DPI (other than DISKUS), n	2	N/A

All data are mean values unless otherwise stated.

*Qualitative scale: 1 = least severe, 10 = most severe.

^†^Inhaler used to deliver maintenance therapy.

COPD = chronic obstructive pulmonary disorder; DPI = dry powder inhaler; HFA = hydrofluoroalkane (propellant); MDI = metered dose inhaler.

### Findings from interviews: key attributes

Participants reported high levels of satisfaction with and a very positive experience of using the ELLIPTA DPI. Each of the key attributes of the DPI, as targeted by the specific questions asked, were perceived positively by the majority of participants. The findings of the content analysis of the responses to these questions, together with representative quotations for each relevant emergent theme and cluster, are provided in Table 2. The most frequently encountered themes included simplicity of action, speed and ease of operation, fit of the inhaler to the hand and mouthpiece to the lips, and dose count awareness.

**Table 2:** Key attributes of the ELLIPTA DPI: selected quotations from participants with asthma (N = 33) and COPD (N = 42)

**Relevant emergent themes (no. of COPD/asthma patients reporting)**	**Relevant quotations to illustrate theme, COPD patients**	**Relevant quotations to illustrate theme, asthma patients**
**Ease of use**		
Ease and simplicity (16/17)	“Life made less complicated”	“It’s straight-forward in its use”
	“Very easy”	“You really don’t have to think about it, it’s that easy”
	“Extremely easy to use”	“It a no brainer in how to use”
Simple/Intuitive (9/5)	“It’s simple and takes the frustration out of it”	“It has a very simple design”
	“It’s easy to learn to use”	“It is really well thought out”
		“Really don’t need instructions to use the device, it’s very intuitive.”
	“You don’t need to be a rocket scientist to use the device, it’s really very simple”	
Simple steps (8/3)	“Just flip open the cover and inhale”	“Open and inhale”
	“One hand, one shot, no thought.”	“Pull back the lever and inhale”
Issues with Lever Action (2/5)	“Opening is a little difficult, the cover feels tight when I go to slide”	“Lever drags when I pinch it to slide it open”
		“Sometimes the lever would move back a little when I open it”
**Amount of time to use device**		
Quick/Just seconds (27/21)	“Takes no time at all to use”	“Less time than any other device”
	“I can’t imagine it being any faster to use”	
	“3 to 4 seconds”	
	“Only 3 seconds to use, it’s so easy”	“About the same amount of time as my current pump”
		“No one will notice I’m using it, it’s so quick. I’m always on the go and this is a good thing”
		“It’s incredibly quick and fast”
Number of steps (5/6)	“Open, inhale and close”	“Flip, inhale and rinse…you’re done”
	“Just a flip and you breathe it in”	“Just cock it, breathe and go”
**Hand feel of the device**		
Fits hand (17/13)	“I have a small hand and it fits it well”	“Fits in my hand nicely”
	“It’s the right size and shape to fit my hand”	“It has a substantial feel that I like”
Never slipped (12/12)	“Never slipped from my hand”	“It never slipped so I never dropped it”
		“I don’t recall ever dropping it”
	“It doesn’t slip around when you grab it”	
		“It never slipped from my hand always felt very secure”
Not too large/compact (11/5)	“It’s compact and not bulky”	“It’s not that big, which is great”
	“It’s not too large, which I like”	“It has a compact size that’s good”
Comfortable to hold (7/3)	“The feel in my hand is comfortable”	“It’s comfortable in my hand”
Easy to hold (6/2)	“It’s got a nice curve to it that makes it nice to hold”	“It has a texture that helps me to hold on to it”
Like grip/design ridges (2/4)	“The ridge marks in the thing help me to hold it”	“It has little grippers that I like, ridges; they really help”
**Ease of opening/closing device**		
Easy (9/14)	“It’s easy to open with one hand”	“It’s obvious what to open and how and there is not much resistance to push it open and closed”
	“Just slides open and closed with little effort”	
		“It really takes no strength to slide the cap”
Simple (6/14)	“Just flip it and it’s open, it’s very easy to do”	“Trigger is the cover, it is so easy”
	“It’s just a simple operation, you can even do it with one hand”	“It’s really mistake proof”
		“It’s simple to just pull open. It closes the same way.”
Difficult to slide cover (4/3)	“Sometimes it may stick a little and I need to push a little harder”	“Takes a little pressure to open, was not real smooth”
	“A little harder to open than Advair”	“The lever action was a little gritty from time to time”
No problems (general) (3/3)	“Cover never got stuck, never had a problem”	“No complications, no troubles”
	“There just were no problems”	
Grips/ridges/lip (3/3)	“I can slide it over with my thumb. I use the little raised lip to push it over”	“I like the flare ups on the cover, makes it easier to open and close”
**Feel of device when placed to the lips**		
Fits well/seals well (14/17)	“It’s shaped like my lips so I felt I got a good seal”	“It fits well, there is more of it to fit to my lips”
	“Fit my lips perfectly”	“Easy to make a tight seal to be sure inhaling the medicine, and not air.”
	“It seals better than others and I feel I get more of the medicine. I feel I’m getting the full dose.”	“Fit nicely to my lips”
Comfortable (12/9)	“It felt right”	“It was comfortable, I didn’t have to purse my lips”
	“It felt really good to hold it up to my lips”	“Just very comfortable”
	“It felt very comfortable”	
Placement of lips (3/2)	“I feel it is a little too small or short, I need to get my mouth around it.”	“The mouthpiece is a little shallow, I put my mouth further up”
**Ease of activation**		
Works even with small breath (14/5)	“Worked even when my breath was shallow”	“I had no problem breathing in the medicine on days I could breathe, it was effortless”
	“It was not hard to draw out the medicine”	
	“I didn’t feel I needed to suck as hard”	“I think it takes less breath to get the medicine in”
	“One big inhale and I know I got the medicine”	
Easy (4/11)	“Less difficult than an MDI”	“Almost too easy, I called my doctor to make sure I was doing it right”
	“Very easy to inhale”	“It was just real easy to do”
Medicine delivery (7/7)	“I could feel the medicine. I could feel the powder going in”	“I could feel the air flowing through so I knew something was happening”
	“I just felt like I got all the medicine”	“Felt like I could feel the medicine and it was easy to direct it to the back of the mouth”
Not propelled (0/4)		“I would like it better if it was propelled”
		“Don’t feel it the same way as a pump. I have no proof it’s working or dispensing.”
**Use and value of dose counter**		
Big/easy to see/read (17/19)	“I can see the red indicator even without my glasses”	“The black and white numbers are very easy to see”
	“I can read the numbers without my glasses”	“I can see them without my glasses”
	“I don’t need to pull out my magnification to see the numbers”	“The numbers are big and easy to read”
	“It’s large enough I can see it”	
Keeps me on track (12/6)	“Indicates to me when doses are getting low”	“Keeps me on track with my medicine”
	“Makes it easier for me to keep up and not forget”	“Helps me know if I took my medicine each day
	“It’s easy to read. It tells me when to refill. And, I know if I took my medicine”	“I like to know if I missed a day, and this helps me
Red indicator (8/7)	“The red indicator tells me I’m close to being out of medicine”	“The red strip tells me when I’m low”
	“9 or less and it goes to red and tells me I’m getting close to being out of medicine”	“I like that it turns red when getting low, I know to reorder.”
	“I like the red indicator”	
Helpful (6/6)	“It has actual numbers and that’s helpful, no guessing”	“It’s simply helpful”

### Ease of use

Patients had been briefed at the start of the clinical study by a member of staff at the clinical study site on how to use the ELLIPTA DPI, often with the aid of a demonstration inhaler. When asked about their use of the instruction leaflet, interview participants reported that they had not needed to refer to instructions or ask follow-up questions after having been shown how to use the inhaler. During the free response segment of the interview (prior to the rating of key attributes), several participants spontaneously reported on the straightforwardness and intuitiveness of the use of the DPI, describing the few steps required (“open and inhale, that’s it: not much to it”) and the time taken to use the ELLIPTA DPI (“a matter of seconds to use”). These sentiments were reflected by the findings of the content analysis of the underlying reasons provided for rating of the ease of use of the ELLIPTA DPI, and amount of time taken to use the inhaler (Table 2). Participants with experience of other DPI use commented on the similarity of the technique used to operate the ELLIPTA DPI to that of inhalers such as the DISKUS™.

### Dose counter

The dose counter of the ELLIPTA DPI helps to provide the user with confirmation that a dose has been delivered from the DPI after they have opened and closed the cover. Participants reported that the dose counter made it easy to stay on track with their medication, and provided an indication of the need to refill. Participants with poor eyesight also reported that the dose counter was clearly readable, without the need for corrective lenses or magnification. The red indicator (which displays when 9 or fewer doses remain in the DPI) was praised by several participants as providing a visual reminder to renew their medication.

### Cover

Most participants, particularly those who had previous experience of using a metered dose inhaler (MDI), expressed a positive opinion of the cover (or ‘lid’) of the ELLIPTA DPI, which is opened in order to activate a dose and uncover the mouthpiece. This cover remains closed when not in use, protecting the mouthpiece, and is therefore considered to be hygienic. The most frequently encountered content themes in relation to the cover were easy to open and close (several patients commented that they could open it with one hand), and simple to operate, with a raised lip which can be slid up using the thumb. MDI users noted that it is useful that the cover remains attached to the body of the inhaler, as opposed to a separate cover which has to be removed and replaced. Some participants remarked that the cover dragged, or sprang back slightly when pushed open, but it was also noted that the inhaler would not open inadvertently (e.g. during transit), thereby wasting a dose, and that it was possible to partially open the DPI without using a dose. Three participants with asthma and four with COPD did report inadvertently actuating the DPI during the clinical trial by opening and closing the cover when they were not ready to inhale a dose, resulting in the loss of the dose. This was typically due to becoming distracted and accidentally opening the cover when not wishing to inhale a dose. No participant reported more than one error resulting in loss of a dose during the clinical trials.

### Inhalation

Delivery of medication from the ELLIPTA DPI is achieved by first opening the cover to actuate the dose, then inhaling the medication from the mouthpiece with a long, steady, deep breath. The mouthpiece of the ELLIPTA DPI was reported to be comfortable and well-shaped and to create a good seal with the lips during use. Several participants commented that the mouthpiece increased their confidence that they were receiving a full dose of the medication relative to other inhalers they were currently using. Inhalation itself was described as easy. A frequently-encountered response theme, particularly among participants with COPD, was that the medication can be drawn out of the DPI with a shallow breath. Many participants described being able to feel the powder while inhaling, which provided confirmation that they had received a dose, although some asthma patients expressed preference for a propelled inhaler. A small minority of participants with experience of using an MDI or HandiHaler™ felt that the mouthpiece of the DPI was too short/small.

### Handling and storage

Most participants reported finding the handling of the ELLIPTA DPI to be comfortable and intuitive. Participants commented positively on the ergonomics of the inhaler, with the DPI’s fit to the hand, grip and compact size emerging as frequently cited themes. Most participants used two-handed methods to open and close the cover, either pinching the cover between thumb and forefinger or pulling it down with the thumb. A few patients with larger hands used a one-handed method of opening the DPI. Some participants with COPD and arthritis (seven in total) reported experiencing some difficulties with the opening and closing of the DPI during worsening of arthritis symptoms. However, these participants also reported that the ergonomic design and ridged cover meant that they were able to slide open the ELLIPTA DPI without having to grip the cover. In addition, the ridged side grips were considered by participants to decrease the likelihood of the inhaler slipping from the hand. In terms of storage, participants reported that the ELLIPTA DPI is small enough to be carried in a pocket or purse, and that the flat base aids the storage of the inhaler by enabling it to stand unsupported.

### Preference relative to other inhalers

A summary of participants’ preference for the ELLIPTA DPI relative to other inhalers is provided in Table 3. The majority of participants with asthma and most participants with COPD preferred the ELLIPTA DPI to the inhaler(s) used to deliver their current medication (as prescribed after the end of the clinical study from which they were recruited).

**Table 3:** Summary of patient preference for the ELLIPTA DPI relative to specified other inhalers

**Comparator device**	**No. of patients using comparator device**	**No. (%) of patients expressing preference for the ELLIPTA DPI**
**Total**		
DISKUS	42	33 (79)
MDI/HFA	30	23 (77)
HandiHaler	20	19 (95)
**Asthma**		
DISKUS	21	15 (71)
MDI/HFA	10	6 (60)
**COPD**		
DISKUS	21	18 (86)
MDI/HFA	20	17 (85)
HandiHaler	20	19 (95)

COPD = chronic obstructive pulmonary disorder; DPI = dry powder inhaler; HFA = hydrofluoroalkane (propellant); MDI = metered dose inhaler.

The outcomes of inductive content analysis performed on the reasons given by patients for their preference are provided for asthma patients (Table 4) and COPD patients (Table 5).

**Table 4:** Findings of inductive content analysis of inhaler preference responses of interview participants with asthma

**Relevant emergent themes (no. of patients reporting)**	**Clusters within theme (no. of raw mentions)**	**Relevant quotations to illustrate theme**
**A – DISKUS (N = 21)**		
Simpler and one less step (13)	Ease of use (5)	“Simply open, inhale, and close”
		“Easy and fool proof”
	Less steps or one step (4)	“One less movement, don’t need to slide that lever over”
		“It’s one less step”
	Simplicity of use (4)	“Simple, we have enough complexity in our lives”
		“Intuitive, easy to figure out how to use”
Size and ease of handling (10)	Smaller, more compact (7)	“I like the size, it’s smaller”
	Fits hand / grip (5)	“Fits in my hand more comfortably”
		“I like the grooves so it’s not slippery”
Portability and storage (9)	Carries / packs well (8)	“Small, it fits in my shirt pocket”
		“Easy to carry in a pocket”
	Stores well (4)	“It’s easy to store because it stands up”
		“It takes up less space”
Mouthpiece (8)	Better seal, ensures delivery (6)	“With this mouthpiece I don’t worry about getting my medicine”
		“With lip seal I feel I’m getting the full benefit of the medicine”
	Comfortable size/shape (3)	“The shape makes the mouthpiece more comfortable”
		“Mouthpiece is ergonomically designed”
Counter (7)	Size (5)	“I like the size of the numbers”
		“Main reason is the big counter”
	Easy to see (3)	“I don’t have to squint to see the numbers”
Color (4)	Bland / dislike (3)	“The color doesn’t stand out”
	Diskus is better color (1)	“Purple is easier to find and helps me to remember to use”
**B – MDI (N = 10)**		
Coordination (8)	No timing inhalation (4)	“I don’t have to worry about timing”
		“I have more control over getting the medication”
	Does not propel the medication (4)	“It doesn’t shoot out the medicine”
		“I like the propellant because it pushes the medicine where it needs to be”
Mouthpiece (5)	Good fit / good seal (3)	“Fit my mouth well”
		“Fit well”
	Mouthpiece size (2)	“It’s not a bulky size”
Ease of Use (5)	Ease (4)	“Very easy to use”
		“Well thought out to make it easy”
	Intuitive (1)	“Very easy and obvious what to do”
Dose counter (4)	Plan and keep track of doses (2)	“Counter helps me to plan ahead”
	Easy to read counter (2)	“I like the counter is better, it’s bigger and easier to read”
One piece cover (3)	One piece construction (2)	“Cover is attached and I can’t lose it”
	Sanitary (1)	“Covers the mouthpiece to keep it clean”

Patients who were using A) DISKUS, B) MDI to deliver their current medication.

**Table 5:** Findings of inductive content analysis of inhaler preference for interview participants with COPD

**Relevant emergent themes (no. of patients reporting)**	**Clusters within theme (no. of raw mentions)**	**Relevant quotations to illustrate theme**
**A – DISKUS (N = 21)**		
Mouthpiece seal and security of getting all the medication (15)	Security of getting all my medication (8)	“I know I’m getting a good seal and all the medication”
		“I’m more confident the medication gets where it’s supposed to go”
		“With this mouthpiece I know I’m not going to suck air”
	Mouthpiece fit/seal to lips (6)	“Mouthpiece fits the shape of my mouth”
		“I like the mouthpiece and how it fits my lips”
	Mouthpiece comfort (2)	“Mouthpiece was more comfortable”
	More airflow (1)	“I found it easier to inhale, I got more air flow”
Ease & comfort to hold and operate (13)	Easy/better grip (8)	“Has grip lines and makes it easier to hold”
		“The bigger top is easier to grip”
	Fits well in hand (3)	“More slim, it fits nicely in my hand”
	Can operate with one hand (3)	“Easier to flip open and can do it with one hand”
	More comfortable to hold (2)	“Feels comfortable when it’s in my hand”
Simple, less steps to operate (13)	One less step—saves time (8)	“One motion, no extra steps to open”
		“One less step”
	Easier/simpler to use/user friendly (7)	“Easier, no extra step”
		“85% easier to use”
Counter makes it easy to read dose (10)	Easy to see/read/don’t need glasses (5)	“Can glance at it and see the dose count”
		“I don’t need to put on my glasses to see it”
	Larger counter (4)	“Like it because it has a larger dose counter”
	Informs me when low (2)	“Gives me knowledge of when I’ll run out of medicine”
Ease of storage (4)	Ability to stand (4)	“It’s easy to store because it stands up”
**B – HandiHaler (N = 20)**		
Simple mode of action / Fewer steps (17)	Simpler / not complex (11)	“Takes a lot less time to use”
		“This is much simpler because I have arthritis in my hands”
	Fewer steps / less time (8)	“I reach in my bag, grab the new device, open it, breathe and put it back; it’s much less time”
		“Just takes less time”
	Difficulties managing Handihaler (4)	“Spiriva is more difficult, many more steps to manage and more messy”
Fewer components (7)	No capsules (5)	“Capsules are a pain in the butt”
		“Because there is no pill there is no complication”
	No blister pack (3)	“I don’t need a scissors to open the package that has the pill inside, which is good because I have arthritis”
Reliable (6)	More assured getting medicine (4)	“I wonder if I’m getting all the medication out of the capsule, and here I don’t have to worry.”
	Less chance for error (4)	“Less chance for mistakes—this is fool-proof”
		“Much less chance of a mistake”
**C – MDI/HFA (N = 20)**		
Ease of operation (8)	No shaking/no priming (3)	“I don’t have to shake it, I like that.”
	One puff, not two (3)	“It’s easier because I only have to take one puff, instead of two”
	Fewer steps (2)	“It’s just fewer steps to take”
Coordination (7)	Ease of operation (3)	“It’s easy because I have arthritis and I could pull down the cover (on ELLIPTA), it can be difficult to push down on the other device”
	No coordination (2)	“It’s simpler to do because there’s no timing to worry about”
Dose counter (4)	Has a counter (2)	“I like that it has a counter, I don’t think mine does”
	Easy to read counter (2)	“The counter is bigger and I don’t see as well, so this is good for me”
One piece cover (4)	Better cover (4)	“This is a better mouthpiece cover, it is much more secure”

HFA = hydrofluoroalkane (propellant); MDI = metered dose inhaler.

Patients who were currently using A) DISKUS, B) HandiHaler, C) MDI/HFA to deliver their current medication.

The ELLIPTA DPI was preferred to DISKUS in over three quarters of comparisons made by 42 participants with asthma or COPD. The main drivers of preference were the simplicity and ease of use of the ELLIPTA DPI; its ease of handling; size; portability and storage; the shape of the mouthpiece and seal made with the lips on inhalation; and the size and visibility of the dose counter. The mouthpiece seal and ease of handling were the most prominent response themes among participants with COPD, whereas the simplicity of operation and storage and portability of the inhaler were more frequently cited by asthma patients.

The ELLIPTA DPI was preferred to current MDI by over three quarters of participants with asthma or COPD. The most frequently encountered themes underlying preference for ELLIPTA over MDI included the ease, speed and simplicity of operation (in particular, that there is no need to shake or prime the inhaler before use) and that co-ordination of activation of the DPI with inhalation is not required; the reduced number of inhalations needed; the presence of a readable dose counter; the security and hygiene of the ELLIPTA DPI’s cover; and the comfort and fit of the mouthpiece.

Assessing COPD participants who were using the Handihaler at the time of the study, all but one participant preferred the ELLIPTA DPI. The main driver of preference was the simplicity of the mode of action of the ELLIPTA DPI compared with that of the HandiHaler, without the need to open a blister pack and insert a capsule into the device ahead of inhalation; and patients’ increased confidence that they received a complete dose of medication based on the contoured mouthpiece and simplicity of the device design and operation.

### Performance scores

Indicative performance scores for the ELLIPTA DPI were recorded on a scale of 1 (worst) to 10 (best) for nine separate attributes of the DPI. The scores obtained using this method are provided to aid the interpretation of participants’ perceptions of the inhaler that is supported by the textual context provided, and should be viewed with caution in light of the small sample sizes (42 patients with COPD; 33 patients with asthma) from which they are derived.

**Figure 1:**
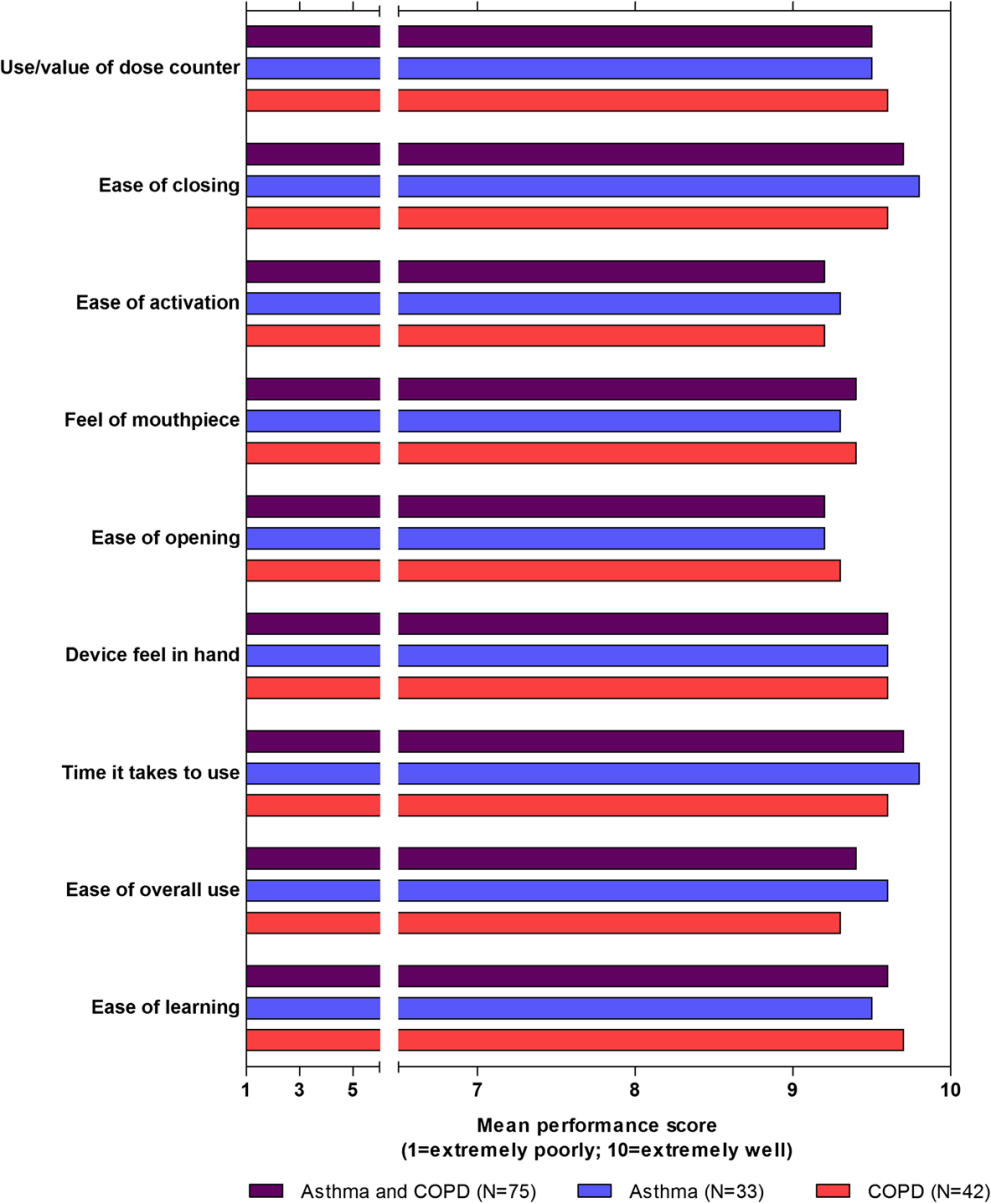
**Overall performance scores.** COPD = chronic obstructive pulmonary disorder.

**Figure 2:**
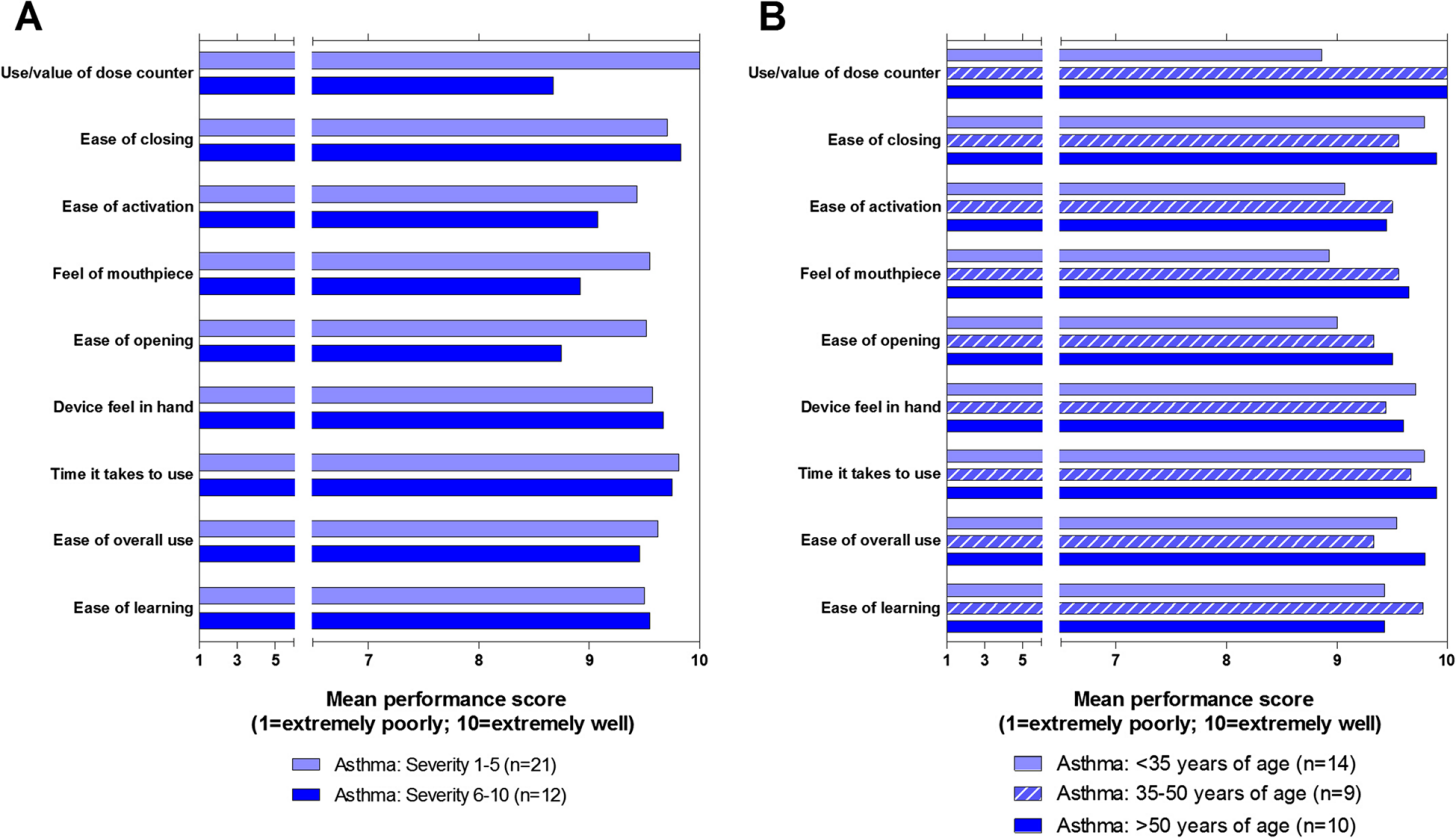
**Asthma performance scores by subgroup defined by: A) asthma severity; B) age.** Note: asthma severity scores in **A** range from 1 (not at all severe) to 10 (extremely severe).

**Figure 3:**
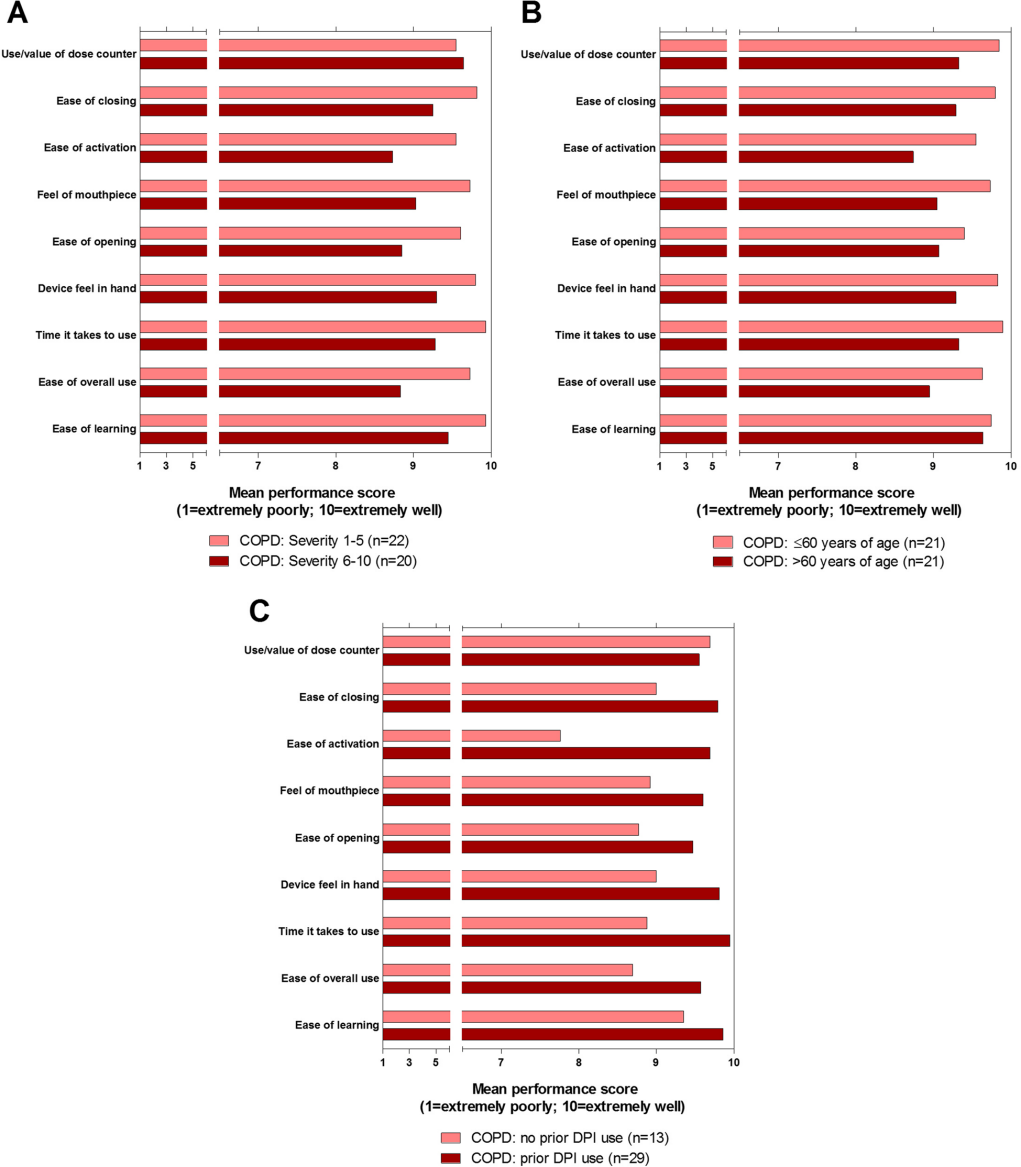
**COPD performance scores by subgroup defined by: A) COPD severity; B) age; C) prior DPI use.** Note: COPD severity scores in **A** range from 1 (not at all severe) to 10 (extremely severe). COPD = chronic obstructive pulmonary disorder; DPI = dry powder inhaler.

Average performance scores in participants with asthma and COPD were >9 for all nine attributes (Figure 1). Average performance scores in subgroups of participants with asthma (Figure 2A–B and Figure 3A–C), following stratification by age, severity, duration of diagnosis, and (COPD only) previous DPI use, were >8 in all but one subgroup-category combination. The only score of <8, for the ease of activation attribute, was recorded in the subset of participants with COPD who did not have previous experience in the use of a DPI.

## Discussion

### Summary of study findings

This qualitative in-depth interview-based study was carried out in a subset of patients (n = 75) who had previously completed clinical trials of a novel ICS/LABA maintenance therapy for asthma and COPD. It was designed as an exploratory study that aimed to investigate patient perception of attributes of the ELLIPTA DPI in an inductive manner, and to gather comparative information versus other inhalers that had also been used by the participants. Attributes of the ELLIPTA DPI that were viewed positively by participants included ease of use and simplicity of operation, the visibility and ease of interpretation of the dose counter, the feel and fit of the inhalation mouthpiece, and design ergonomics. No attribute of the inhaler was viewed negatively overall. Participants with asthma and COPD expressed preference for the ELLIPTA DPI compared with the inhaler they were using at the time of the study. It should, however, be borne in mind that the circumstances in which participants were introduced to and used the ELLIPTA DPI, within the context of a randomized controlled trial, inevitably contrasted with their usage of established inhalers in routine clinical practice.

### Inhalers, adherence to medication, and treatment outcomes in asthma and COPD

The inhaler through which medication is delivered has been recognized as central to the clinical effectiveness of any inhaled therapy [8,17]. The ability of the patient to use the inhaler correctly is therefore an important factor in ensuring that the therapy is effectively delivered to the lung [21]. Suboptimal inhaler technique is associated with reduced control in asthma and COPD [10] and addressing incorrect inhaler technique has been the subject of calls to action in asthma [22,23] and interventional trials in COPD assessing the best means of educating patients on inhaler use [24]. The easier and more straightforward an inhaler is to use, the lower the likelihood of inhaler error [22]. COPD patients tend to be older on average than patients with asthma, more likely to be regularly taking multiple medications, and more likely to have physical or cognitive impairments that may impact their ability to use inhalers correctly [4].

In addition to correct inhaler technique, adherence is also a key factor in determining treatment outcomes [16,25]. Inhaler choice has been hypothesized to play an important role in influencing whether or not patients adhere to their prescribed therapy. In asthma, the use of a DPI to deliver maintenance ICS has been found to be associated with improved adherence compared with MDIs [26]. Adherence to inhaled treatment has previously been reported to be significantly associated with dosing frequency [27], inhaler technique [28], and patient satisfaction with the inhaler [9], although it should be noted that the association between satisfaction and adherence is unclear [29,30].

Ease of use and handling of the inhaler are important factors in determining patient satisfaction [31]. The physical difficulty of handling and using medications has previously been found to be associated with suboptimal adherence to COPD medication regimens [32]. In both asthma and COPD, inhaler characteristics associated with patient satisfaction include those described in the present study, namely those pertaining to ease of use such as simplicity of operation, lack of multiple steps, and ease of learning [33,34].

Because the ELLIPTA DPI has no real-world test experience and has only been used within the confines of randomized controlled trials, there are no data in the current scientific literature about patient perception of the inhaler. The airflow resistance of the ELLIPTA DPI has however been extensively studied *in vitro*. In a quantitative study, using an Electronic Lung™ breathing simulator [35] to replicate patient-representative inhalation flow rates, the delivery performance of the DPI was investigated and showed consistent powder delivery across a wide range of inhalation parameters. These data support the suitability of the ELLIPTA DPI for use across a wide range of disease states, from mild asthma to severe COPD [36]. Patients were required to demonstrate correct handling and use of the ELLIPTA DPI to continue in the clinical trials from which interview participants were recruited. As such, inhaler technique was not explored during the present study. Data on patient competence in use of the DPI, as assessed by study investigators during three trials of FF or FF/VI, are reported separately [37]. Ninety-five percent of patients used the DPI correctly after one demonstration of its usage at randomization.

### Study strengths and weaknesses

The results of this exploratory, inductive study, including the performance scores, are suitable for qualitative interpretation. The nature of the study, and the small number of patients who participated in the interviews, meant that statistical analysis of or inference from the attribute ratings and preference findings would not have been appropriate. As a retrospective study, it was not possible to perform a real-time assessment of patient preferences.

The participants in this study were patients with asthma or COPD who had used the ELLIPTA DPI in a clinical trial setting and were based in the USA; as such, the patient group may not have been truly representative of the wider patient population. As a result of the eligibility criteria for most of the trials from which participants were drawn, all of the participants had previous experience of using at least one other inhaler, and almost all participants were currently prescribed inhaled controller medication. In view of the possibility of recall bias, inhaler preference comparisons versus the ELLIPTA DPI are reported only for the currently prescribed inhaler. The asthma and COPD participant populations were adequately representative of the broader population in terms of age.

Patients with lower motivation may be less likely to have volunteered for the clinical trials than those who attended all clinic visits and completed a trial (and were therefore eligible to participate in this qualitative study). It is also possible that any patients having a strong dislike of the ELLIPTA DPI may not have completed the trial and would thereby be ineligible for participation in the qualitative research. Study data were anonymised; as such, only patient-reported information on the severity of asthma or COPD was available, which may limit the interpretation of the effect of disease severity on inhaler preference. Finally, as the ELLIPTA DPI was used to deliver treatment to patients in clinical trials, proper instruction in correct use of the inhaler was provided, and patients who were unable to use the DPI correctly were withdrawn from the trial and would therefore not have participated in the interviews; however, the incidence of inability to use the DPI, reported separately, has been observed to be low [37]. The ease of DPI use reported here may not be fully realized in real-world clinical practice if adequate instruction in correct technique is not provided [38].

## Conclusion

The ELLIPTA DPI was preferred over current inhalers by the majority of asthma and COPD patients recruited from phase III studies to participate in the post-study interviews. Ease of use, simplicity of operation and the design of the mouthpiece were the most frequently encountered response themes underlying preference. Whether these findings from interviews of clinical trial participants will translate to clinical practice is an important research question that warrants further study.

## Abbreviations

COPD: Chronic obstructive pulmonary disease; DPI: Dry powder inhaler; FF: Fluticasone furoate; ICS: Inhaled corticosteroid; LABA: Long-acting β_2_ agonist; MDI: Metered dose inhaler; VI: Vilanterol.

## Competing interests

**MWW** is employed by Strategic Eye, Inc. who carried out this qualitative study on behalf of GlaxoSmithKline. **HS**, **KG**, and **RW** are employed by and hold stock in GlaxoSmithKline. **PD** is a former employee of GlaxoSmithKline.

## Authors’ contributions

**HS** participated in the analysis and interpretation of the data and provided scientific guidance for the paper. **PD** participated in the conception and design of the study and the analysis and interpretation of the data. **KG** participated in the interpretation of the data. **RW** contributed to the acquisition, analysis and interpretation of the data. **MWW** participated in the conception and design of the study, the acquisition of data and the analysis and interpretation of the data. All authors had access to all of the data, were involved in every stage of the preparation of the manuscript and approved the final version for submission. The sponsor did not place any restriction on authors about the statements made in the final paper.

## Pre-publication history

The pre-publication history for this paper can be accessed here:

http://www.biomedcentral.com/1471-2466/13/72/prepub

## Supplementary Material

Additional file 1: Table S1 Details of phase III clinical trials from which interview participants with A) COPD and B) asthma were recruited. Click here for file

Additional file 2 Asthma discussion guide. Click here for file

Additional file 3 **Visual stimuli used in patient interviews.** The following visual stimuli were provided to subjects: (**A**) photos of the unlabelled (blank) ELLIPTA DPI as used in the phase III studies, (**B**) patient instructional directions, (**C**) visual prompts illustrating ways in which patients have been observed to open the ELLIPTA DPI in previous research, (**D**) visual prompts illustrating ways in which patients have been observed to hold the ELLIPTA DPI during inhalation in previous research. Click here for file
