# Supplementary material for: Qualitative assessment of attributes and ease of use of the ELLIPTA™ dry powder inhaler for delivery of maintenance therapy for asthma and COPD
Source: BMC Pulm Med. 2013 Dec 7;13:72. doi: 10.1186/1471-2466-13-72 (PMC4029771; doi:10.1186/1471-2466-13-72)
Supplement: Additional file 1: Table S1 — Details of phase III clinical trials from which interview participants with A) COPD and B) asthma were recruited. [file 1471-2466-13-72-S1.docx]

**Supplementary tables**

**Table S1 Details of phase III clinical trials from which interview participants with A) COPD and B) asthma were recruited**

**A)**

| **Study number** | **Study medications** | **Study primary objective** | **Study duration (weeks)** | **Medication used during run-in** | **Medication used prior to screening** |
| --- | --- | --- | --- | --- | --- |
| HZC102871 and  HZC102970 | FF/VI 50/25 mcg, FF/VI 100/25 mcg, FF/VI 200/25 mcg and VI 25 mcg once daily via the two-strip DPI | Annual rate of moderate/severe COPD exacerbations | 52 | Open-label FP/SAL 250/50 mcg twice daily via DISKUS, 4 weeks | No pre-screening medication use requirement. 68–74% of patients used ICS; 63–69% LABA; 35–35% tiotropium (delivery device unspecified) |
| HZC112206 | FF/VI 50/25 mcg, FF/VI 100/25 mcg,  FF 100 mcg, VI 25 mcg and placebo once-daily via the two-strip DPI | Treatment differences in weighted mean 0–4 h FEV_1_ on day 168 and trough FEV_1_ on day 169 | 24 | Single-blind placebo via ELLIPTA DPI, 2 weeks | No pre-screening medication use requirement. 23% of patients used ICS; 32% used LABA; 26% used tiotropium (delivery devices unspecified) |
| HZC112207 | FF/VI 100/25 mcg, FF/VI 200/25 mcg,  FF 100 mcg,  FF 200 mcg,  VI 25 mcg and placebo once-daily via the two-strip DPI | Treatment differences in weighted mean 0–4 h FEV_1_ on day 168 and trough FEV_1_ on day 169 | 24 | Single-blind placebo via ELLIPTA DPI, 2 weeks | No pre-screening medication use requirement. 24% of patients used ICS; 35% used LABA; 24% used tiotropium (delivery devices unspecified) |

**B)**

| **Study number** | **Study medications** | **Study primary objective** | **Study duration (weeks)** | **Medication used during run-in** | **Medication used prior to screening** |
| --- | --- | --- | --- | --- | --- |
| HZA106827 | FF/VI 100/25 mcg,  FF 100 mcg and placebo via the two-strip DPI | Change from baseline in trough FEV_1_ and weighted mean FEV_1_ after 12 wks | 12 | Stable dose of same ICS used during 4 weeks prior to visit 1; LABA not permitted; rescue albuterol allowed,  4 weeks | ICS use for ≥12 weeks prior to visit 1, with stable low-mid dose ICS (FP 100–250 mcg twice daily or equivalent) or low-dose ICS with LABA for ≥4 weeks prior to visit 1, delivery device unspecified |
| FFA114496 | FF 200 mcg and  FF 100 mcg via the two-strip DPI | Change from baseline in trough pre-bronchodilator FEV_1_ after 24 wks | 24 | Stable dose of baseline ICS medication; non-corticosteroid controllers not permitted (rescue albuterol allowed), 4 weeks | Stable regimen of mid-high dose ICS required for at least 4 weeks prior to visit 1, delivery device unspecified |

COPD = chronic obstructive pulmonary disorder; DPI = dry powder inhaler; FEV_1_ = forced expiratory volume in 1 second; FF = fluticasone furoate; FP = fluticasone propionate; ICS = inhaled corticosteroid; LABA = long-acting β_2_ agonist; SAL = salmeterol; VI = vilanterol.
