# Supplementary material for: Qualitative assessment of attributes and ease of use of the ELLIPTA™ dry powder inhaler for delivery of maintenance therapy for asthma and COPD
Source: BMC Pulm Med. 2013 Dec 7;13:72. doi: 10.1186/1471-2466-13-72 (PMC4029771; doi:10.1186/1471-2466-13-72)
Supplement: Additional file 2 — Asthma discussion guide. [file 1471-2466-13-72-S2.docx]

**Qualitative assessment of attributes and ease of use of the ELLIPTA™ dry powder inhaler for delivery of maintenance therapy for asthma and COPD**

Henrik Svedsater, Peter Dale, Karl Garrill, Richard Walker, Mark W. Woepse

**Additional file 1**

**Asthma discussion guide**

**1. Introduction and warm-up (10 minutes)**

Welcome interviewee and introduce moderator.

Honest answers; no vested interest, etc.

Audio-taping.

**For phone/web interviews, confirm that subject is logged on to the website. For phone/mail interviews, confirm presence of sealed envelopes.**

As you know, we have invited you today because you have identified yourself as someone who has participated in a clinical research study using a dry powder inhaler (DPI; ‘the study device’). I’d like to talk with you about that device and understand more about it from your perspective.

**Determine if the subject has any visual impairments and/or conditions that affect his/her hand strength, and therefore might affect their ability to easily operate the device.**

First, some general questions…

1. Do you have asthma? How long have you suffered from it? Overall, how severe would you say your asthma is on a 10-point scale? (10 = extremely severe; 1= not at all severe).
2. What medications are you taking to treat your asthma? **Important to get a full list of devices used by the subject.**
3. I’d like to ask you a few questions about the device in which the asthma medication that you’re using right now comes in. I am not interested in what you think of the medicine, I only want you to think about the device. How satisfied are you with your current device and how it works? Why is that?

**Ask subject to refer to their current asthma device(s) to lend added depth/insight into their comments**.

1. Are there any other asthma medication delivery devices that you have used? Explain.
   What was your experience with that device? What, if anything, did you like about that device? Dislike? Why?

**2.** **Device discussion (25 minutes)**

**Throughout the entire dialogue, be certain to emphasise the specific type/name of device being discussed to reduce subject confusion.**

Do you recall using a DPI device (‘the study device’) during the recent clinical study you just completed? I would like to ask you some questions about your use of that device. I also want you to keep in mind that I am not interested in the medicine itself, but only the device.

1. What was your reaction to this device when you first saw it? Why? What was your reaction after you had an opportunity to use it? Why? Did your impression of the device change after you used it? How? What is your most vivid memory about use of the device itself?
2. How would you rate this device in terms of learning how to use it? On a scale of 1 to 10, where 10 means it was extremely easy to learn to use, and 1 means it was extremely difficult to learn to use, what number would you choose? Why?
3. An information leaflet was provided to you during the clinical trial in which you participated that explained how to use the device… do you recall that leaflet? Did you read that leaflet? Why/why not?

**If subject is familiar with/read the leaflet, ask:** How would you rate the patient information leaflet in terms of explaining how to use the inhaler? Rate the leaflet 1 to 10, where 10 means it is extremely easy to understand the leaflet and 1 means it is extremely difficult to understand. Why do you rate it as such?

1. What, if anything, did you like about using the study device? Why?
2. What, if anything, did you not like about using the study device? Why?
3. How did the study device compare to the devices you use now?
   Why do you say that? **Probe advantages/disadvantages, including handling, ease of use, etc**.

If the medicine that has been prescribed by your doctor and that you are currently using were available both in the study device and the one you are currently using, tell me, which device would you choose? Why? One main reason?

**Repeat for any other devices subject is currently using or has used in the past that they recall clearly.**

1. Where did you store the device? Why there? What was it like to store the device?
2. While you were using it, did you ever carry or transport the device with you? Why did you do this?

***For face-to-face sessions:* show study device to aid discussion.**

***For phone/web interviews:* instruct subject to refer to their computer screen to view the study device.**

***For phone/mail interviews:* instruct subject to refer to the letter coded envelopes and open only when instructed.**

**If necessary we would allow the subject to view the device earlier in the interview if they require help remembering.**

**Ask them to show/explain how they handled and used the device, from opening through storage. Determine if the subject appears to understand how to use the device correctly/is following the proper steps for use (aid as necessary with pictures for phone/web and mail interviews).**

1. What do you feel are the advantages, if any, of using this device versus what you use now?
2. What do you feel are the disadvantages, if any, of this device compared to what you now use?
3. Using a 10-point scale where 10 means it performs extremely well and 1 means it performs extremely poorly, how would you rate this device in terms of:
   - - Ease of use overall
     - Amount of time to use the device
     - Hand feel of the device
     - Ease of opening the device
     - Feel of device when placed to the lips
     - Ease of activation
     - Ease of closing the device
     - Use and value of the dose counter.

**(Discuss reasons for each rating).**

# 3. Device follow-up probes (10 minutes)

1. When you first received the device did you happen to slide it open to have a look without realising that you had primed the device and wasted a dose? How did you feel about that? Did it ever happen again? What, if anything, would you suggest be done or shared to help prevent this from happening?
2. Show me/explain to me the grip that you have found to be best for you in opening the device. Do you know if this is the grip that is shown in the instruction leaflet for opening the device, or is this a different grip that you have found to be more comfortable? R**efer to pictures for phone/web or mail interviews.**
3. When opening the cover to give yourself a dose of medication, did you hear a ‘click’ at the end of the stroke when opening the cover? What, if anything, did that mean to you?
4. Did you ever look at the dose counter and find that it was halfway in counting – showing a transition between two numbers? How often did this occur? **If yes:** What was your reaction to this when you saw it?
5. When using the device, was there a particular way in which you always held the device? Explain. **(Probe if held with 1 or 2 hands, with which fingers, etc.)**

Was there a side of the device that you thought was the top or upside of the device?

**(If needed, probe position of the device when held, i.e. if subject held device with dose counter side up, at an angle, etc.)**

Did you always hold the device that way? Explain. **If subject is familiar with patient instructions, ask if they tried or used the approach for holding the device outlined in the instructions and ask if they found it comfortable and why.**

1. Anything that you would want them to change about the study device? Why?
2. **Present device usage instructions:** Looking again at these patient instructions, what, if anything, do you find unclear or difficult to understand? Anything missing that would have been helpful? What? Why? How, if at all, would you improve these instructions?
   Anything that you feel is not needed or is confusing within the instructions? Explain.

**If subject mentioned any problems in 3A, above, ask how that issue/problem could have been better addressed or called out in the instructions.**

1. What would you tell someone who has asthma about this device if they have never seen it? Why?

**Thank and remind subject about confidential nature.**

**Chronic obstructive pulmonary disorder (COPD) discussion guide**

**1. Introduction and warm-up (10 minutes)**

Welcome interviewee and introduce moderator.

Honest answers; no vested interest, etc.

Audio-taping.

**For phone/web interviews, confirm that subject is logged on to the website. For phone/mail interviews, confirm presence of sealed envelopes.**

As you know, we have invited you today because you have identified yourself as someone who has participated in a clinical research study using a dry powder inhaler (DPI; ‘the study device’). I’d like to talk with you about that device and understand more about it from your perspective.

**Determine if the subject has any visual impairments and/or conditions that affect his/her hand strength, and therefore, might affect their ability to easily operate the device.**

First, some general questions…

1. Do you have COPD? How long have you suffered from it? Overall, how severe would you say your COPD is on a 10-point scale? (10 = extremely severe; 1= not at all severe).
2. What medications are you taking to treat your COPD? **Important to get a full list of devices used by the subject.**
3. I’d like to ask you a few questions about the device in which the COPD medication that you’re using right now comes in. I am not interested in what you think of the medicine, I only want you to think about the device. How satisfied are you with your current device and how it works? Why is that?

**Ask subject to refer to their current COPD device(s) to lend added depth/insight into their comments.**

1. Are there any other COPD medication delivery devices that you have used? Explain. What was your experience with that device? What, if anything, did you like about that device? Dislike? Why?

# 2. Device discussion (25 minutes)

**Throughout the entire dialogue, be certain to emphasise the specific type/name of device being discussed to reduce subject confusion.**

Do you recall using a DPI device (‘the study device’) during the recent clinical study you just completed? I would like to ask you some questions about your use of that device. I also want you to keep in mind that I am not interested in the medicine itself, but only the device.

1. What was your reaction to this device when you first saw it? Why? What was your reaction after you had an opportunity to use it? Why? Did your impression of the device change after you used it? How? What is your most vivid memory about use of the device itself?
2. How would you rate this device in terms of learning how to use it. On a scale of 1 to 10, where 10 means it was extremely easy to learn to use and 1 means it was extremely difficult to learn to use, what number would you choose? Why?
3. An information leaflet was provided to you during the clinical trial in which you participated that explained how to use the device… do you recall that leaflet? Did you read that leaflet? Why/why not?

**If subject is familiar with/read the leaflet, ask:** How would you rate the patient information leaflet in terms of explaining how to use the inhaler? Rate the leaflet 1 to 10 where 10 means it is extremely easy to understand the leaflet and 1 means it is extremely difficult to understand. Why do you rate it as such?

1. What, if anything, did you like about using the study device? Why?
2. What, if anything, did you not like about using the study device? Why?
3. How did the study device compare to the devices you use now? Why do you say that? **Probe advantages/disadvantages, including handling, ease of use, etc.**

If the medicine that has been prescribed by your doctor and that you are currently using were available both in this device and the one you are currently using, tell me, which device would you choose? Why? One main reason?

**Repeat for any other devices subject is currently using or has used in the past that they recall clearly.**

1. Where did you store the device? Why there? What was it like to store the device?
2. While you were using it, did you ever carry or transport the device with you? Why did you do this?

***For face-to-face sessions:* show study device to aid discussion.**

***For phone/web interviews:* instruct subject to refer to their computer screen to view the study device.**

***For phone/mail interviews:* instruct subject to refer to the letter coded envelopes and open only when instructed.**

**If necessary we would allow the subject to view the device earlier in the interview if they require help remembering.**

**Ask them to show/explain how they handled and used the device, from opening through storage. Determine if the subject appears to understand how to use the device correctly/is following the proper steps for use (aid as necessary with pictures for phone/web and mail interviews)**

1. What do you feel are the advantages, if any, of using this device versus what you use now?
2. What do you feel are the disadvantages, if any, of this device compared to what you now use?
3. Using a 10-point scale where 10 means it performs extremely well and 1 means it performs extremely poorly, how would you rate this device in terms of:
   - - Ease of use overall
     - Amount of time to use the device
     - Hand feel of the device
     - Ease of opening the device
     - Feel of device when placed to the lips
     - Ease of activation
     - Ease of closing the device
     - Use and value of the dose counter.

**(Discuss reasons for each rating).**

# 3. Device follow-up probes (10 minutes)

1. When you first received the device did you happen to slide it open it to have a look without realising that you had primed the device and wasted a dose? How did you feel about that? Did it ever happen again?

What, if anything, would you suggest be done or shared to help prevent this from happening?

1. Show me/explain to me the grip that you have found to be best for you in opening the device. Do you know if this is the grip that is shown in the instruction leaflet for opening the device, or is this a different grip that you have found to be more comfortable? **Refer to pictures for phone, web or mail interviews.**
2. When opening the cover to give yourself a dose of medication, did you hear a ‘click’ at the end of the stroke when opening the cover? What, if anything, did that mean to you?
3. Did you ever look at the dose counter and find that it was halfway in counting – showing a transition between two numbers? How often did this occur? **If yes:** What was your reaction to this when you saw it?
4. When using the device, was there a particular way in which you always held the device? Explain. **(Probe if held with 1 or 2 hands, with which fingers, etc.)**

Was there a side of the device that you thought was the top or upside of the device? **(If needed, probe position of the device when held, i.e. if subject held device with dose counter side up, at an angle, etc.)**

Did you always hold the device that way? Explain. **If subject is familiar with patient instructions, ask if they tried or used the approach for holding the device outlined in the instructions and ask if they found it comfortable and why.**

1. Anything that you would want them to change about the study device? Why?
2. **Present device usage instructions:** Looking again at these patient instructions, what, if anything, do you find unclear or difficult to understand? Anything missing that would have been helpful? What? Why? How, if at all would you improve these instructions? Anything that you feel that is not needed or is confusing within the instructions? Explain.

**If subject speaks to any problems in 3A, above, ask how that issue/problem could have been better addressed or called out in the instructions.**

1. What would you tell someone who has COPD about this device if they have never seen it? Why?

**Thank and remind subject about confidential nature**
