# Supplementary material for: Qualitative assessment of attributes and ease of use of the ELLIPTA™ dry powder inhaler for delivery of maintenance therapy for asthma and COPD
Source: BMC Pulm Med. 2013 Dec 7;13:72. doi: 10.1186/1471-2466-13-72 (PMC4029771; doi:10.1186/1471-2466-13-72)
Supplement: Additional file 3 — Visual stimuli used in patient interviews. The following visual stimuli were provided to subjects: (A) photos of the unlabelled (blank) ELLIPTA DPI as used in the phase III studies, (B) patient instructional directions, (C) visual prompts illustrating ways in which patients have been observed to open the ELLIPTA DPI in previous research, (D) visual prompts illustrating ways in which patients have been observed to hold the ELLIPTA DPI during inhalation in previous research. [file 1471-2466-13-72-S3.docx]

**Additional file 2**

**Visual stimuli used in patient interviews**

The following visual stimuli were provided to subjects: (**A**) photos of the unlabelled (blank) ELLIPTA DPI as used in the phase III studies, (**B**) patient instructional directions, (**C**) visual prompts illustrating ways in which patients have been observed to open the ELLIPTA DPI in previous research, (**D**) visual prompts illustrating ways in which patients have been observed to hold the ELLIPTA DPI during inhalation in previous research.

**A.**


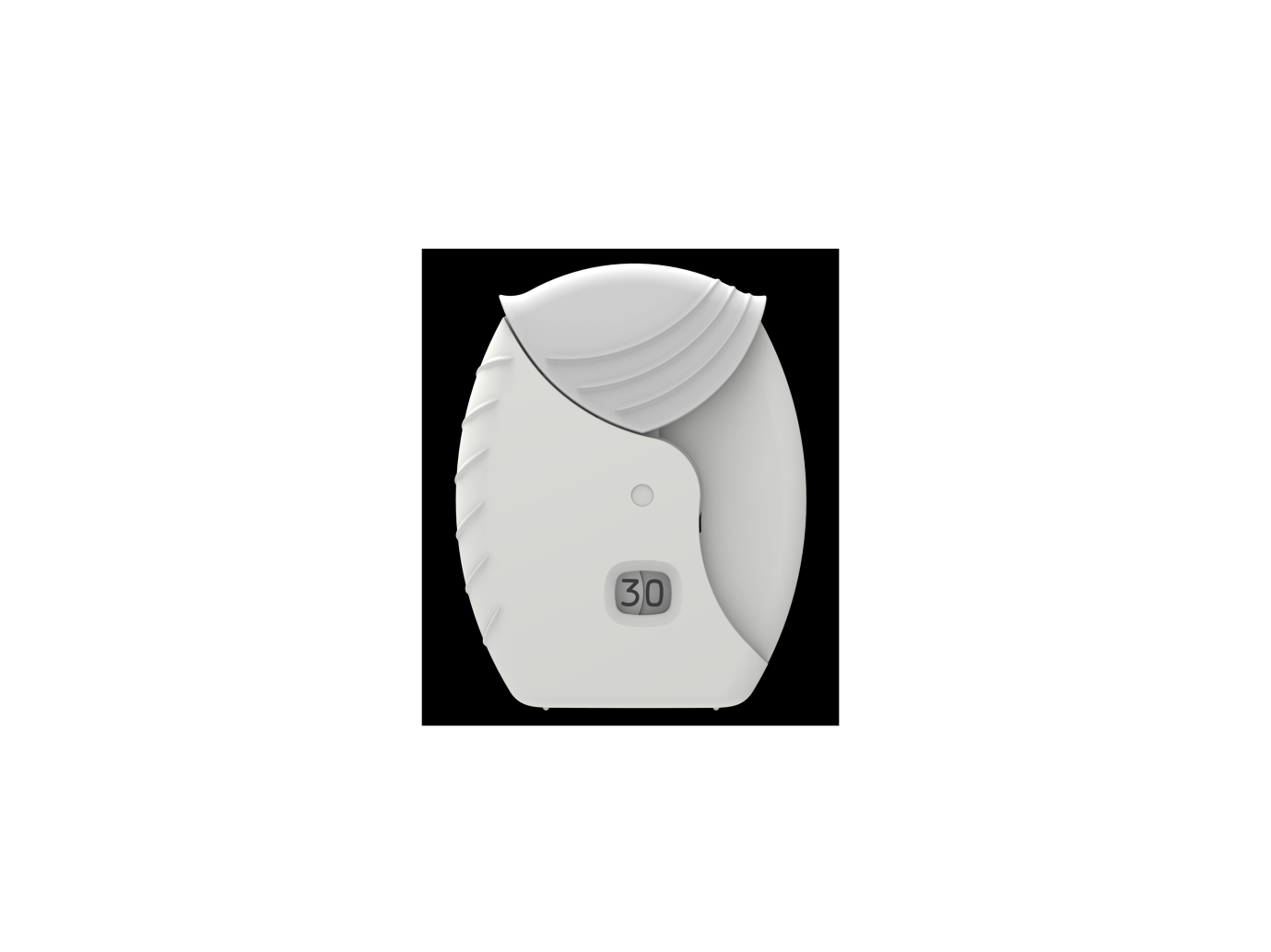


**B.**


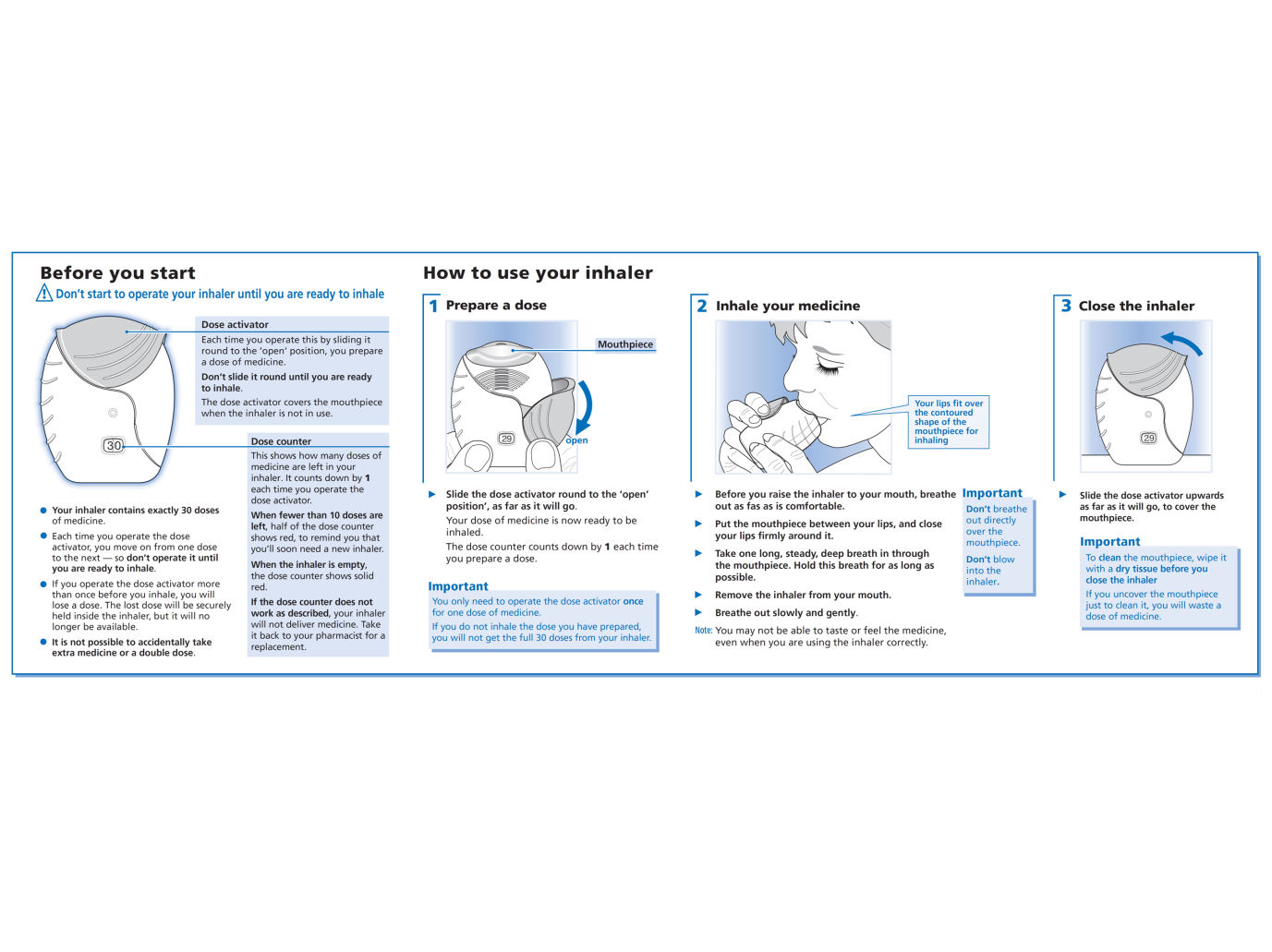


Note: Draft version for illustrative purposes only.

**C.**


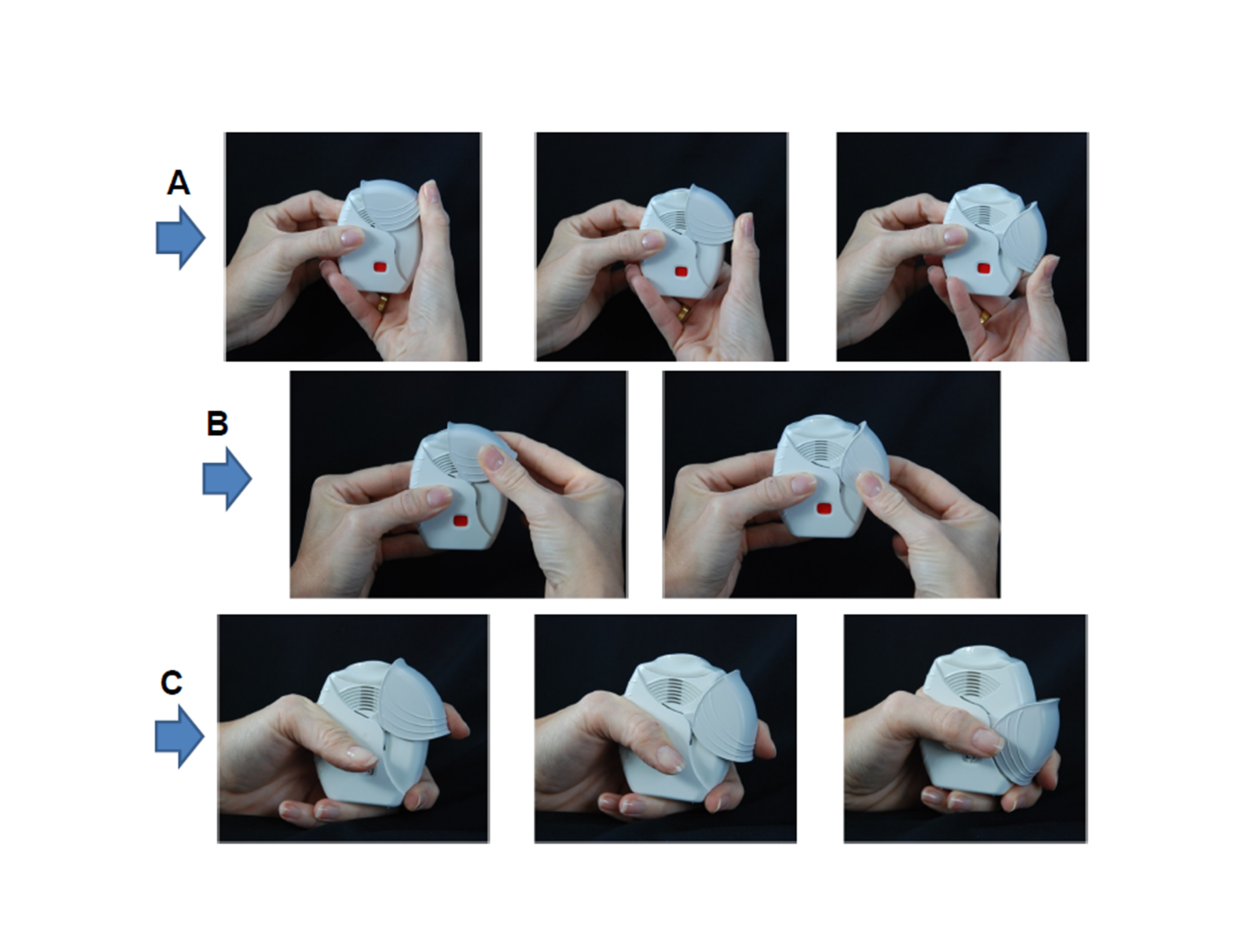


**D.**

**
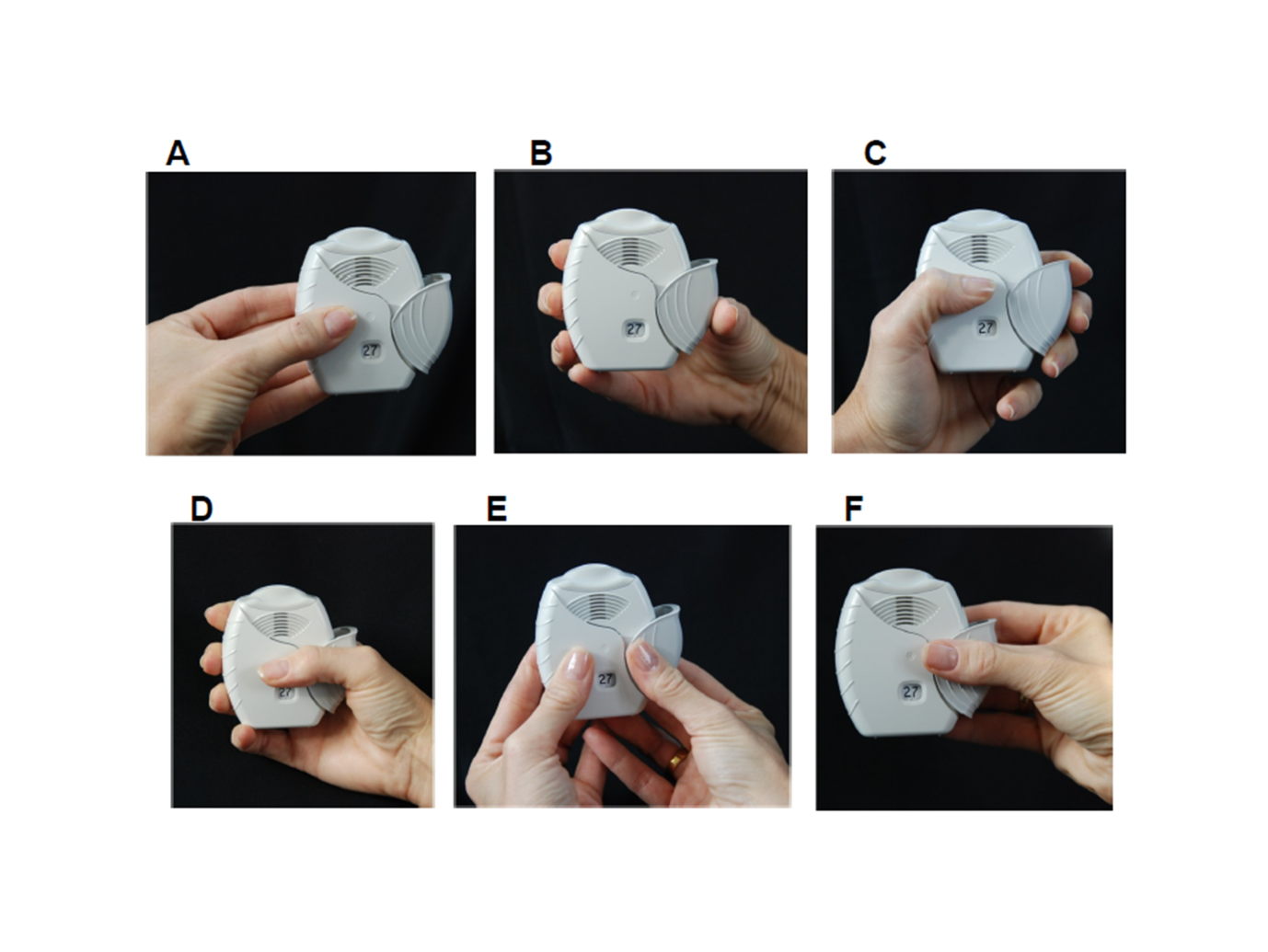
**
